# A Critical Evaluation of Patient Pathways and Missed Opportunities in Treatment for Heart Failure

**DOI:** 10.3390/jcdd9120455

**Published:** 2022-12-12

**Authors:** Chun Shing Kwok, Duwarakan Satchithananda, Fozia Z. Ahmed, Colin D. Chue, Diane Barker, Ashish Patwala, Simon Duckett, Christian D. Mallen

**Affiliations:** 1 Department of Post-Qualifying Healthcare Practice, Birmingham City University, City South Campus, Birmingham B15 3TN, UK; 2 Department of Cardiology, University Hospitals of North Midlands NHS Trust, Stoke-on-Trent ST4 6QG, UK; 3 Department of Cardiology, Manchester University NHS Foundation Trust, Manchester M13 9PL, UK; 4 Department of Cardiology, University Hospitals Birmingham NHS Foundation Trust, Birmingham B15 2WB, UK; 5 School of Medicine, Keele University, Keele ST5 5BG, UK

**Keywords:** heart failure, treatments, therapy, opportunities

## Abstract

Background: Heart failure (HF) is a global problem responsible for significant morbidity and mortality. Methods: This review describes the patient pathways and missed opportunities related to treatment for patients with HF. Results: The contemporary management strategies in HF, including medical therapies, device therapy, transplant, and palliative care. Despite the strong evidence base for therapies that improve prognosis and symptoms, there remains a large number of patients that are not optimally managed. The treatment of patients with HF is highly influenced by those who are caring for them and varies widely across geographical regions. HF patients can be broadly classified into two key groups: those who have known HF, and those who are incidentally found to have reduced left ventricular systolic dysfunction or other cardiac abnormality when an echocardiogram is performed. While all patients are under the care of a general practitioner or family doctor, in other instances, non-cardiologist physicians, cardiologists, and specialist HF nurses—each will have varying levels of expertise in managing HF—are part of the broader team involved in the specialist management of patients with HF. Conclusions: There are many potential missed opportunities in HF treatment, which include general opportunities, medications, etiology-specific therapy, device therapy, therapies when initial treatments fail, and palliative care.

## 1. Introduction

Heart failure (HF) is a global problem responsible for significant morbidity and mortality. It is predicted that with an aging population and improved survival from acute myocardial infarction, patients with HF will continue to increase [1,2]. Being potentially life-changing, HF can cause distressing symptoms and physical disability for the patient, with a significant impact on families. It can be the catalyst for the transition from independent living with no physical restrictions to reduced functional capacity and a need for assistance with daily activities. For some patients, this can translate into depression and poor quality of life [3]. The enormity of the problem has prompted major research on treatments for HF over the last few decades. This has culminated in contemporary management strategies that include medical therapies, device therapy, advanced HF therapies such as transplantation and durable left ventricular assist device therapy, palliative care, general opportunities, as well as the need for monitoring, preventative care, and care coordination, which are supported by both American and European guidelines [4,5]. This arsenal of treatments has transformed the care of patients with HF, and many patients are living longer, with good quality of life. Despite the multitude of therapies available, there are still patients that receive care that does not adhere to guidelines or care that could have been improved. To better understand the care that patients receive, and those who may be overseeing care, it is important to consider the patient pathway from diagnosis to treatment.

## 2. Definition of Heart Failure

HF is a common clinical syndrome that causes symptoms due to structural or functional cardiac disorders that impair the ability of the ventricle to fill with or eject blood [4]. The pathology underlying the HF syndrome may be a consequence of diseases of the myocardium, pericardium, endocardium, heart valves, or vessels, or of metabolic disorders. HF due to left ventricular dysfunction is categorized according to left ventricular ejection fraction (LVEF) into HF with reduced ejection fraction (with LVEF ≤40 percent, known as HFrEF; also referred to as systolic HF), HF with preserved ejection fraction (with LVEF ≥50 percent; known as HFpEF; also referred to as diastolic HF), and HF with mildly reduced ejection fraction (with LVEF 41 to 49 percent; known as HFmrEF).

## 3. Missed Opportunities in Heart Failure Treatment

### 3.1. Consideration of Patient Pathways

Patient pathways are a new approach to considering what may happen to patients with conditions, the care that they receive, and their outcomes. This approach is important considering that pathways may be important to evaluate quality of care [6] and also be a means to audit care practices [7] that may be relevant for clinicians, health services, and also companies [8]. The patient pathway is defined by the series of clinically relevant events that take place for a patient, which involves a combination of activities of the patient and their interaction and care that they receive from health services [9]. The patient pathway review is a method that utilizes clinical expertise to define the possible paths for patients [9]. This methodology has been applied to chest pain [10], atrial fibrillation [11], and COVID-19 [12]. Most recently, the patient pathways for the diagnosis of heart failure have been described, where the pathway framework is applied to consider what happens to patients up to the point of diagnosis. As the misdiagnosis of heart failure is well documented in the literature [13], the consideration of the pathways and missed opportunities has been used to evaluate what might happen to patients up to the point of diagnosis of HF [14]. However, this approach has not been used for pathways related to the treatment of HF once the diagnosis is made.

### 3.2. Pathway of Care in Heart Failure

There are two pathways wherein patients who have HF and those at risk of HF may be identified. As defined in the recent universal definition, HF is a clinical syndrome with symptoms and signs caused by a structural and/or functional cardiac abnormality and corroborated by elevated natriuretic peptide levels and or objective evidence of pulmonary or systemic congestion [15]. An important consideration here is that patients must have clinical features of the HF syndrome, since there is also a population of patients who are incidentally found to have structural and/or functional cardiac abnormalities or elevated natriuretic peptide levels without symptoms or features of congestion. In addition to the recent consensus definition for HF, there have also been modifications to the classification. To draw attention to the different stages of HF, the ACC/AHA describe four stages of HF: At Risk (stage A), Pre-HF (stage B), HF (stage C), and Advanced HF (stage D) [4].

**Figure 1:**
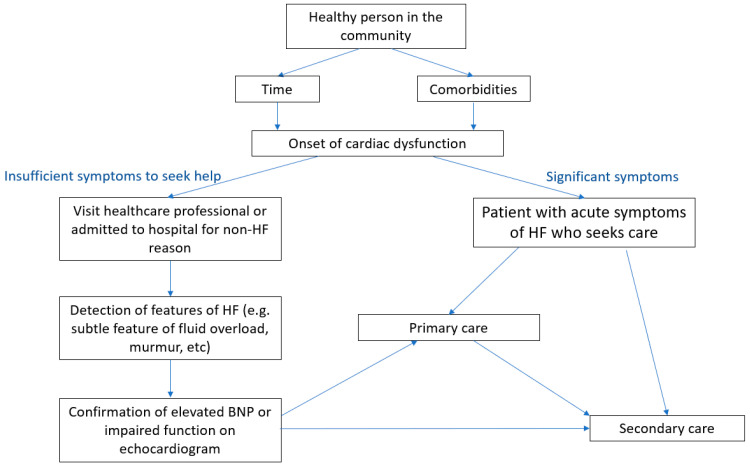
Pathways for population with heart failure and those at risk of heart failure.

For patients with significant symptoms (stages C and D), the pathway is well established and embedded in existing clinical care pathways, whereby patients will seek healthcare professionals either in primary or secondary care. The other group of patients with “Pre-HF” but not symptomatic may be found to have an elevated BNP or structural abnormality on echocardiography [16]. They may be found in any setting, such as a routine primary care visit or inpatient stay for other medical or surgical problems. In either path, the end points will lead to primary care or secondary care evaluation. These two paths are outlined in Figure 1.

**Figure 2:**
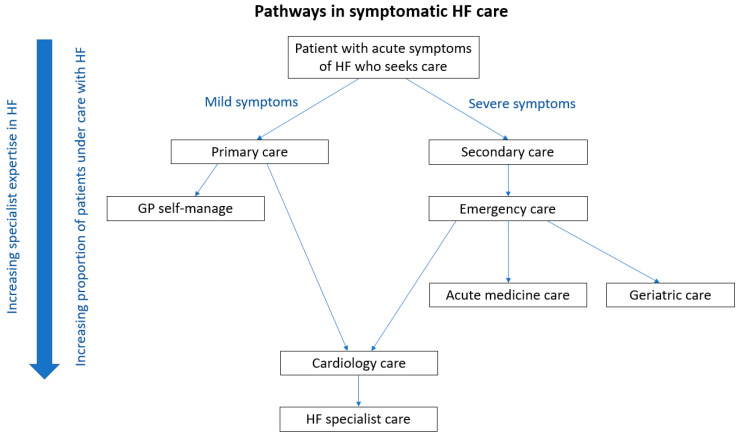
Pathways for care for patients with symptomatic heart failure.

To appreciate the opportunities for better care in the treatment of HF, it is important to recognize those who may be involved in the diagnosis and treatment of HF along the patient pathway (Figure 2). The widespread availability of brain natriuretic peptide testing, CXR, and transthoracic echocardiography has improved the assessment of patients with suspected HF.

Depending on the severity of symptoms, most clinicians will recognize the combination of dyspnea, orthopnea, fatigue, and peripheral edema together with an elevated jugular venous pressure and basal lung crepitations as potential HF and order these tests. Once identified, HF may be treated directly in the community by physicians or referred to hospital for specialist input. The alternative pathway for patients is based on severe symptoms such that they present acute to emergency services with decompensated HF. They will then either be managed by the acute medical team or cardiology specialists as inpatients. Some of the elderly patients may further go on to receive care from geriatric teams. While all inpatient teams will eventually treat the congestion with diuretics in decompensated HF, whether or not patients will have other missed opportunities in treatment is variable. It is important to be aware that although patients present with symptoms of HF, a significant number are not treated for HF. The incorrect treatment is highlighted in the National Confidential Enquiry in Patient Outcomes and Death report in the UK for acute HF as antibiotic therapy, which was used in nearly half of patients [17]. There are strong data to indicate that HF patients managed on cardiology wards have higher prescriptions of guideline-recommended HF treatments and lower rates of inpatient mortality compared to those managed on other wards. Similarly, 1-year mortality is lowest among those who receive cardiology follow-up post-discharge [18].

However, HF is a leading cause of cardiovascular bed occupancy in the UK, and in view of this consideration, it is not feasible for all patients to be accommodated on cardiology wards, though this does not preclude specialist input. Specialist inpatient HF team input (known as HF in reach) has been shown to be associated with improved use of evidence-based therapies and patient outcomes for mortality [19]. National HF audit data from the United Kingdom suggest that in-hospital mortality is lower for patients with access to specialist care (7.9%) compared to those who do not have access (13.3%) [20], so the fact that patients are cared for by other professionals or based on non-cardiology wards does not preclude access to specialist care.

### 3.3. Who Provides Long-Term Care for Patients with Heart Failure

**Figure 3:**
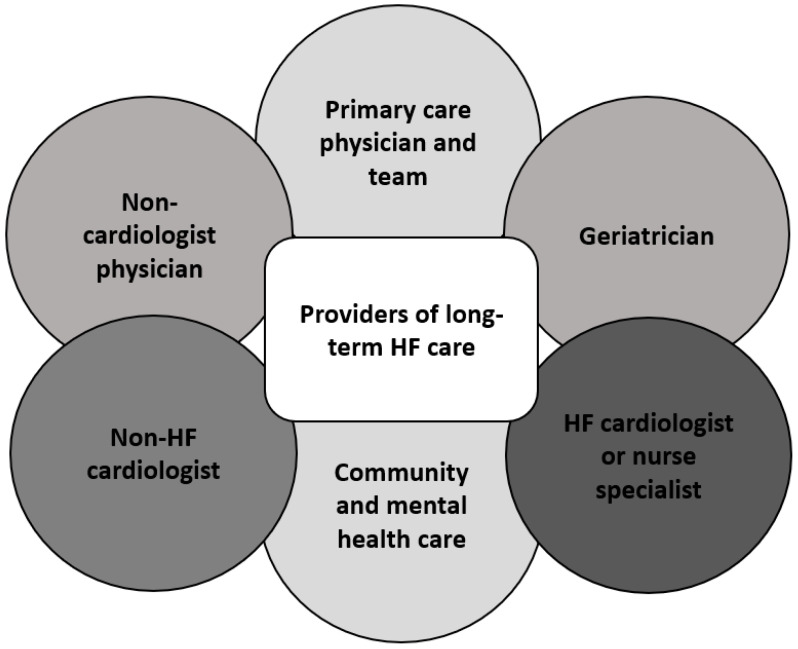
Providers of long-term heart failure care.

HF management is a team-based approach that starts at diagnosis with follow-up visits, which is multidisciplinary with different teams. The reality is that not all HF patients are managed primarily by a HF specialist, and there are also inequalities in care related to patients with HFmREF and HFpEF, as not all HF services cater to these patients. This is in part historical, due to the fact that there has been a paucity of evidence-based treatments indicated for use in patients with ejection fractions >40%. Complex elderly patients who are frail may also end up under the primary care of a geriatric specialist. Equally, especially in more rural settings, patients with HF may be cared for by their primary care physician alone, because it is challenging to attend specialist centers. This is particularly true with the advent of telemedicine, where there can be easy contact between non-specialists and specialists. The professionals who are central in the care of patients with HF are shown in Figure 3. Finally, it is important to highlight that while there is broad recognition that patients with HFrEF should be managed by specialist HF teams, those with HFmREF and HFpEF often have reduced access to specialist HF services. In many instances, community HF services are restricted to those with reduced EF. The reasons for this are multifactorial (limited evidence-based interventions in this group, limited resources prioritized to those with reduced EF), but the limited provision of resources for those HF patients with higher ejection fractions highlights inequalities in the provision of care—often determined on the basis of ejection fraction rather than clinical need.

### 3.4. Missed Opportunities in Heart Failure

Missed opportunities are incidents where reflection on clinical practice would suggest that a better outcome for patients could have been achieved had different actions been taken. Central in this concept is that it is retrospective and takes place in a real-world setting. It forces those involved to consider what happens to patients and determine if different actions could have changed eventual outcomes. This is the ultimate strategy for experiential learning from clinical practice. It also requires unequivocal demonstration that outcomes could have been better in some way (e.g., avoidance of adverse events or cost savings to healthcare provider) if actions deviated from what had taken place. To understand how more patients can benefit from the treatment of HF, it is important to explore the missed opportunities. These missed opportunities in HF can be broadly divided into those that are related to specialist care, general HF care, specific to the etiology of HF, treatments for left ventricular systolic dysfunction, therapies when initial therapies fail, and palliation. It is important that a comprehensive approach is taken in evaluating missed opportunities, where care for patients is considered holistically along the patient pathway, rather than merely opportunities related to primary or secondary care.

### 3.5. Missed Opportunities in Specialist Care

**Figure 4:**
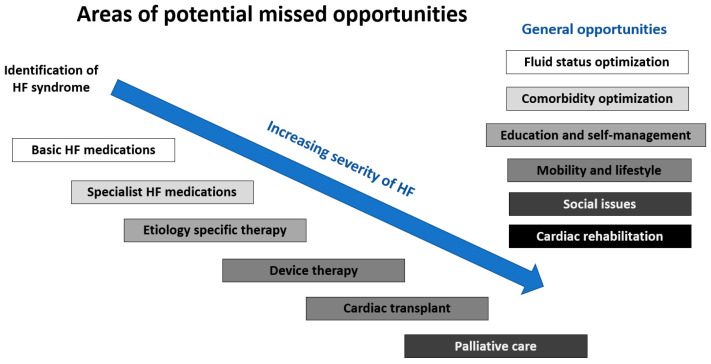
Missed opportunities in the treatment of patients with heart failure.

There are two key advantages to specialist care for patients with HF. The first is that specialists are familiar with up-to-date guidelines and are ideally placed to oversee disease-specific care for patients. Even if they lack facilities for further advanced care such as transplants or pulmonary hypertension management, they will have knowledge of pathways by which to refer patients for advanced therapies and be accustomed to the joint management of complex patients. Non-specialists, who do not primarily manage patients with HF, may be less familiar with recently approved treatments and guidelines or have limited direct access to referral pathways for advanced care. In particular, recent advances in HF pharmacotherapies, including medications such as sacubitril/valsartan [21] and sodium-glucose cotransporter-2 (SGLT2) inhibitors [22], are new treatments whose prescription often requires either specialist initiation or initiation in primary care following specialist recommendation. SGLT2 inhibitors can be used for both HFrEF and HFpEF. Similarly, the implantation of cardiac resynchronization therapy devices [23] and ICDs [24] is only performed in hospital care settings. The second benefit of specialist input is specialist knowledge and access to dedicated cardiac diagnostics to determine the etiology of even the most challenging cases of HF. The potential missed opportunities in HF treatment are shown in Figure 4.

### 3.6. General Measures and Missed Opportunities

The general measures for the care of patients with HF also include serial follow-up to evaluate clinical status, support for HF including the proper use of medication, evaluation of the response to therapy, and assessment of the potential need for changes in management. Visits should include assessment of the ability to perform activities of daily living; volume status and weight; current use of alcohol, tobacco, illicit drugs, and alternative therapies, as well as diet and sodium intake.

The key distressing symptoms of HF primarily relate to those caused by congestion and low cardiac output. Prolonged congestion is known to be associated with potential harm from damage to the heart other organs. Therefore, delays in diuretic treatment and failure to optimize evidence-based therapies in HFrEF may be a missed opportunity. However, these treatments require careful monitoring and close management, particularly with certain agents. Safe optimization and interval monitoring of therapy in a timely manner is key.

Comorbidity management is also an important part of good HF care. This includes common comorbidities such as hypertension, obesity, chronic obstructive lung disease, renal impairment, atrial fibrillation, anemia, iron deficiency, obstructive sleep apnea, frailty, and diabetes mellitus. There may also be additional needs caused by mental health problems resulting from the HF. Hypertension, for example, can be managed with a combination of lifestyle changes and drug therapy. Weight management is important in HF as patients may suffer from fatigue and physical disability, which increases weight gain beyond fluid retention, and there is growing recognition that the obesity epidemic may be contributing to the rising prevalence of HF, both directly (obesity-related HFpEF) and indirectly. Chronic obstructive pulmonary disease can make it challenging to manage breathlessness in HF, and deterioration in lung disease may cause detrimental changes to the pulmonary vasculature and progression of right HF. Renal dysfunction can be a consequence of cardiorenal syndrome or iatrogenic diuretic or angiotensin-converting enzyme antagonist or angiotensin receptor blocker use. It may also be a consequence of nephropathy from iodine-based contrast during a coronary angiogram or gadolinium from a magnetic resonance imaging scan. Arrhythmias such as atrial fibrillation can cause palpitations and worsening dyspnea, which may require medication and anticoagulation. Iron deficiency anemia can exacerbate some of the symptoms of HF, including breathlessness and fatigue, which can be readily corrected with oral or intravenous iron therapy. Obstructive sleep apnea can result in chronic fatigue as well as irreversible changes to the pulmonary vasculature, which increase mortality and can be managed with continuous positive airway pressure. Diabetes is another important comorbidity that increases the risk of adverse cardiovascular events as well as nephropathy, which can be associated with poor outcomes in HF. Because so many factors need to be considered in comprehensive and holistic care for HF patients, it can sometimes be perceived as complex to manage these patients by non-specialists, but identifying and addressing these general areas represents opportunities to improve patient symptoms and outcomes that should not be neglected.

Cardiac rehabilitation is an important part of general HF care that is defined as a multidisciplinary program that includes exercise training, cardiac risk factor modification, psychosocial assessment, and outcome assessment [25]. There is evidence to suggest that cardiac rehabilitation reduces all-cause and HF-specific hospital admissions and may improve health-related quality of life [26]. However, the referral of patients with HF for cardiac rehabilitation is often missed. The exact reason for this is likely multifactorial, with clinician, patient, and health service-related factors. From the clinician perspective, a potential missed opportunity is failure to refer patients to this service who may benefit from it. This relies on the clinician being aware of the benefits of rehabilitation and persuading patients of the advantages of taking part and adhering to recommendations. From the patient perspective, noncompliance can be an issue as they may feel that they will not benefit from rehabilitation, and, in private healthcare settings, the cost and the need for insurance coverage may influence decisions in attending cardiac rehabilitation. The educational aspect of rehabilitation is important as these programs may improve the patient’s basic understanding of HF, its prognosis, and complications, which could empower them to better look after their health. The health service can have an impact as well on the availability of cardiac rehabilitation as rural areas may not have easily accessible services, which limits the uptake of cardiac rehabilitation programs.

### 3.7. Etiology-Specific Missed Opportunities

The advantage of specialist input is subject matter expertise and the ability to arrange tests to confirm the underlying cause of HF—which may be amenable to specific treatments. The most common cause of HF is ischemic heart disease [27], and invasive or non-invasive coronary angiography may be indicated to assess for significant coronary artery disease. In addition, ischemia testing can also be performed with stress echocardiography, myocardial perfusion imaging, and stress cardiac magnetic resonance imaging, where the latter has the advantage that viability can be determined with late gadolinium imaging. The importance of defining etiology is highlighted by patients with valvular heart failure. In this group of patients, medications may be of limited utility, with surgical interventions being the mainstay of treatment. Over the last decade, the options for treating these patients have developed dramatically with the advent of percutaneous valve replacements and interventions [28,29]. However, these patients require specialist input from multiple clinicians and can be missed if they are not reviewed by a HF specialist. Inflammatory conditions of the heart, such as pericarditis and myocarditis, may be reversible or controlled with immune-modulating medications such as high-dose NSAIDs, colchicine, or corticosteroids in the first instance. Identification of arrhythmias contributing to HF may be amenable to rate control medication or catheter ablation procedures. Specific cardiomyopathies that are identified may be treatable or measures can be taken to minimize the risk of progression. Hypertensive cardiomyopathy may be treated with hypertension. HF secondary to thyroid disease or anemia may be treated once detected. Medications such as anthracycline chemotherapy can cause HF, so monitoring these patients and awareness of the risk of HF in this population may reduce the likelihood of causing long-term harm.

### 3.8. Missed Opportunities for Treatment of Patients with Left Ventricular Systolic Dysfunction

Most of the research over the past few decades on HF focuses on patients with severe left ventricular systolic dysfunction. There is a widespread consensus among guidelines supporting the use of angiotensin-converting enzyme inhibitors or angiotensin-converting enzyme blockers, β-blockers, mineralocorticoid receptor antagonists, and ivabradine. There are also newer agents, such as angiotensin receptor neprilysin inhibitors and SGLT2 inhibitors, which have shown benefit in patients with HF and reduced ejection fraction. Missed opportunities may arise when clinicians fail to prescribe these therapies because they are unaware of them or decide not to prescribe them because of unfamiliarity with them. The clinical course of patients with HF can fluctuate, so they may require close monitoring and treatments may fluctuate over time. As there is a clear benefit for these treatments, it is important to re-instigate them when there may have been an acute deterioration or illness whereby medications needed to be stopped [30]. An example of this may be a patient with HF who is admitted with sepsis, who then stops certain treatments because of their hypotensive or nephrotoxic effects.

### 3.9. Missed Opportunities Related to Device Therapy

Some patients with HF may benefit from device therapy. It has been reported that up to 28% of patients with reduced EF HF may be eligible for cardiac resynchronization therapy (CRT) or an implantable cardioverter defibrillator (ICD), and the benefits associated with these therapies are well established [31]. While ICDs reduce the risk of sudden death in patients with HF, CRT devices have the additional advantage of potentially improving symptoms and quality of life and reduce the risk of adverse events, even in mildly symptomatic patients [32]. Nevertheless, under-implantation of both ICD and CRT devices is a global problem, with some countries reporting that only a third of eligible patients have a complex device [33]. The reasons for this are multifactorial. The lack of awareness of guideline-directed indications for complex devices may be implicated. In private healthcare systems, the willingness of patients to pay for the device, or whether the patient has healthcare insurance that can cover the cost of the device, may be important factors influencing whether they receive the device.

CRT is indicated in patients with HF who remain symptomatic despite the optimal medical therapy, who have severe left ventricular systolic dysfunction (left ventricular ejection fraction <35%) and a broad QRS duration. The prolongation of QRS duration and development of bundle branch blocks in HF may not be present at the initial assessment and develop over time. Therefore, in patients who do not initially fulfill this criterion, an annual electrocardiogram is recommended to screen for the broadening of the QRS duration, to ensure timely identification for device-based therapies where indicated. It would be expected that HF cardiologists and specialist HF nurses would be aware of this option of therapy and screen for ECG changes, but there may be delays or missed opportunities if patients are discharged to the care of their GP. Periodic assessment of LV function by means of echocardiography may also be indicated to assess for changes in ejection fraction that may lead to a change in indication for device-based therapies.

ICDs are implanted to reduce the risk of sudden cardiac death in patients with severely impaired left ventricular systolic function (left ventricular ejection fraction <35%) who may or may not have symptoms. The common population who develop heart failure and may be eligible for primary prevention ICD therapy are patients who have acute myocardial infarction. In these patients, it is recommended that assessment for the appropriateness of ICD takes place at least 40 days after AMI. The time lag between the initial event and potential treatment provides an opportunity to titrate guideline-directed medications that may affect the left ventricular ejection fraction. However, the care of post-MI LVSD patients is not standardized and there are significant variations in practice, with a reliance on healthcare practitioners to refer the patient to another team for specialist assessment. In view of these considerations, delays in accessing the right care team, titrating guideline-directed therapies, and the consideration of ICD in the post-MI population are commonplace—representing an opportunity to improve and align the HF and post-MI care pathways.

The main missed opportunities regarding these therapies are a delay to receiving these therapies or patients not having the opportunities to receive these devices despite being eligible according to guidelines. These devices are implanted by secondary care specialists and may not be accessible easily and quickly for patients who are managed by primary care physicians or cardiology specialists from centers that do not implant devices. Delays to these therapies may result in the progression of the cardiac disease to the extent that the risks of the device may outweigh the benefits, or patients may be too frail or comorbid and may require palliative care.

The key consideration is not only whether there was a missed opportunity or not for the device, but whether there was preventable harm and an avoidable missed opportunity. In some cases, the delay in receiving a device may not translate to harm, but, in other cases, patients may be hospitalized with symptoms or patients may even have a cardiac arrest. If there was a missed opportunity related to a lack of awareness by clinicians, education may have an important role to avoid future missed opportunities. This is different from the case in which clinicians were aware of the benefits of the device and recommended this treatment for the patient, but the patient was not able to afford the device in a private healthcare setting due to the inability to pay and the lack of insurance coverage.

### 3.10. Missed Opportunities When Initial Therapies Fail

Advanced heart failure therapies include heart transplantation and durable left ventricular assist device (LVAD) therapy. Opportunities may be missed when considering the timing of referral for such therapies, and also in the management of patients following heart transplantation or those on durable LVAD support.

In the pre-transplant phase, late referrals for chronic HF may be a consequence of clinicians not recognizing the severity of HF. Recommendations for referral for the consideration of advanced HF therapies outline a number of features indicative of adverse prognosis [34,35]. Delayed referral may result in increased risks in the delivery of such treatments or in patients no longer being suitable for heart transplantation. The presence of significant pulmonary vascular disease (defined as a transpulmonary gradient of >12 or pulmonary vascular resistance of >3 Woods units) as a consequence of sustained pulmonary hypertension due to left heart disease precludes heart transplantation due to high rates of acute right heart failure and mortality after transplantation [36]. In such scenarios, selected patients may be suitable for a durable LVAD as a bridge to transplant candidacy, provided that the right ventricular function is adequate. In the presence of persistent pulmonary hypertension and an increased afterload, right ventricular failure can ensue and, in such situations, durable LVAD therapy can be associated with greater risks of acute or chronic right heart failure. If right heart dysfunction is deemed too severe, durable LVAD therapy may be inappropriate and palliation may be preferred. Long-term left ventricular unloading with durable LVAD therapy can reduce pulmonary pressures and reverse pulmonary vascular disease to a sufficient degree to facilitate heart transplantation. In the United Kingdom, such patients are considered of low priority and would only be offered routine heart transplant listing.

Delayed referral in the setting of chronic HF can also be associated with significant end-organ dysfunction, cardiac cachexia, and deconditioning, all of which increase the risks of transplantation, increase the likelihood of a prolonged critical care stay, and add to post-transplant morbidity, including the possible need for renal replacement therapy. In the event of significant end-organ dysfunction, selected patients may be considered for combined organ transplantation, although this extends the wait for suitable donor organs and adds to the complexity of surgery. In most cases, significant end-organ dysfunction, particularly the presence of liver cirrhosis or end-stage kidney disease, is a contraindication to heart transplantation. The presence of marked cardiac cachexia may also render an individual unsuitable for cardiac surgery and palliation may be more appropriate.

Delayed referral of acute HF and cardiogenic shock can also increase the risks of advanced heart failure therapies and mortality. Cardiogenic shock can occur in the setting of an acute ischemic event, acute decompensation of chronic HF, or a new presentation of a non-ischemic process such as fulminant myocarditis. Mortality is high in these patients and is exacerbated by delayed recognition and early mismanagement. Prolonged acidosis, lactatemia, and worsening end-organ function may necessitate the escalation of treatment beyond inotropes to multi-organ support and short-term mechanical circulatory support. Such treatments are invasive, resource-intensive, and carry significant morbidity. It is imperative that a suitable exit strategy is identified prior to embarking on such therapies, with short-term mechanical circulatory support being used as a bridge to recovery, bridge to transplantation, or bridge to an alternative support strategy once end-organ dysfunction and metabolic disturbances have improved.

There is a continued lack of availability of donor organs worldwide, with the demand exceeding the supply. In the current era, the likelihood of receiving a suitable donor heart on the routine heart transplant waiting list is low, as the majority of donor organs in the UK are offered to patients on the urgent and super-urgent waiting lists. These individuals, stratified by a cardiac index on right heart catheterization of <2 L/min/m^2^, require inpatient treatment with continuous infusions of intravenous inotropes or require short-term mechanical circulatory support to maintain end-organ perfusion and metabolic stability prior to heart transplantation. Patients on the routine heart transplant waiting list therefore risk deterioration and are either palliated, receive a durable LVAD as a bridge to transplantation, or are upgraded to the urgent waiting list and placed on intravenous inotropes. Depending on their size and blood group, these patients potentially remain in hospital for several months until a suitable donor offer is made. Lastly, as highlighted by the COVID-19 pandemic, further restrictions in the availability of resources to facilitate heart transplantation, namely a critical care bed and theater team, may result in missed opportunities, even if a suitable organ is identified. The diversion of resources to manage COVID-19 has resulted in longer waits for patients on transplant waiting lists.

Following heart transplantation or the implantation of a durable LVAD, patients undergo lifelong follow-up at the transplant unit. Patients and their relatives are provided with contact telephone numbers for the transplant team and an emergency hotline that is manned 24 h a day, 7 days a week for urgent advice. There is potential for missed opportunities when patients, families, and clinicians do not access specialist advice or are managed in their local non-specialist center. Following transplantation, patients may have complications related to immunosuppression, graft rejection, or opportunistic infection, all of which may not be fully appreciated outside of a transplant center. Durable LVADs pose additional challenges, including complications such as bleeding, pump thrombosis, driveline infection and right ventricular failure, and difficulties with monitoring due to the lack of pulsatile flow with contemporary continuous flow pumps. Blood pressure must be measured using Doppler and pulse oximetry may be unreliable. For these reasons, it may be preferable for such patients to be transferred to their transplant center for further assessment and management.

### 3.11. Missed Opportunities in Palliation

For many patients with HF, advanced HF therapies may be unsuitable due to the presence of significant comorbidities. However, broader psychosocial issues may also be implicated and include substance use (including smoking), a lack of social support, and psychological morbidity. In these circumstances, continued medical therapy and, ultimately, palliation may be more appropriate. The treatment aims will therefore shift to relieving symptoms and improving quality of life. Palliative care is important so that the patient’s wishes, as well as the family’s input, can be considered in the decision-making processes. Decompensated HF and respiratory distress can cause immense suffering for patients and it can be traumatic for families when patients deteriorate. At the same time, resuscitation may not be in the best interest of patients where their HF or comorbidities are not curable. It is important, where possible, while the patient has the capacity as they deteriorate that they are aware of the severity of their condition and the ceiling of care, which is a clinical decision considering the patient’s and family’s perspective. Delayed palliative input can result in patients undergoing unnecessary resuscitation and treatments that may prolong suffering. Acceptance that their HF may be a terminal event may enable patients to plan where they wish to be and identify any relatives that they wish to contact. Clinically, it is not always easy to achieve appropriate palliative care, as early input may be upsetting for patients and families, but delayed input may be a missed opportunity to avoid harming patients with distressing symptoms and unnecessary treatments. There may be cases whereby patients have the desire to die at home, which can be honored if sufficient time is available to make arrangements. The deterioration in HF can be rapid, and this is not always possible, which may be a missed opportunity. In addition, some patients with HF may not benefit from hospitalization, and there may be a missed opportunity for primary care physicians and community HF nurses to make plans so that they can spend their last days in the community and avoid hospitalization. This may be the case for patients who are bedbound or have dementia or those with very limited quality of life, with high levels of dependence for care if living in nursing homes.

## 4. Discussion

Missed opportunities are not exclusively incidents where guidelines directing therapy are not followed. Recommended treatments in HF medications may not be prescribed for a few reasons. First, patients may develop side effects and the treatment may be thus discontinued. This is particularly important when routine discharge data or follow-up data are collected without consideration of events that take place between diagnosis and evaluation. In the analysis of health records, frequently, reasons for not taking medications may not be recorded. Second, patients may choose not to take medications. This may be because of adverse effects but also because the patients do not want to have them or feel that they do not benefit from taking the medication. The opportunity is missed if, on reflection, the clinician should have considered prescribing the treatment when they did not or they could have better counselled the patients about the importance of taking them. This raises an important consideration of whether therapies should be started all at once or whether they should be prescribed in stages. The advantage of the former approach is that the patients will be on all desired treatments and the need to consider adding new drug is not necessary. However, the disadvantage is that if a side effect were to occur, one would have to determine which medication caused it and it should be reduced in dose or discontinued.

Complex factors influence the extent to which patients receive treatment. First of all, it is important that the clinicians looking after patients with HF are aware of all the different treatments. Some clinicians may only implement treatments that are easily available to them or those that they are familiar with. This may be the case for non-specialists such as the general practitioner, general medical consultant, or geriatrician. There are other factors that influence the extent to which cardiologists, general practitioners, and other clinicians may be aware of treatments and decide to prescribe them. The adoption of new therapies in clinical guidelines, as well as the publicity generated from clinical trials, are important influences on the propensity for clinicians to prescribe medications. In addition, new medications are typically promoted through pharmaceutical representative visits or drugs are advertised by the companies that produce them. They promote their drugs to doctors through visits and sponsoring meetings, which help to educate doctors about these medications. The extent to which these representative contact clinicians are present is variable and dependent on the local setting. It is clear that pharmaceutical and device companies have a vested interest to reduce missed opportunities related to treatment delays as this affects their profits.

A checklist may be a means of reducing the risk of missing opportunities in treatments for HF. Checklists enable a comprehensive and systematic approach in order to ensure that no areas are overlooked. The key with such tools is that it is agreed upon among those using the tool that it is to be implemented and the resources are available in these settings. A comprehensive checklist, for example, may not be feasible for a community physician to implement as they may not have access to all the diagnostic tests needed to manage HF or the expertise to use unfamiliar specialist treatments. Similarly, they should be designed to be appropriate for the setting; a detailed and complex evaluation may not be feasible in a 10-min consultation but could be better implemented in the inpatient setting, where evaluation and actions can be taken over several days.

The reality is that treatment in HF depends on the clinical setting and the resources available. A key consideration is how easy it is to access specialist help as, in some places, it may be very easy to access, while, in others, it can take time to arrange. Therefore, the areas highlighted in the current review are meant to enable local services to consider these important factors if they wish to improve the quality of care for the HF patients that they look after. Important in understanding local services is clinical auditing and service evaluation. By evaluating what happens in real life for patients with HF, it is possible to reflect on any areas in the framework described in the review that could be improved on. Any missed opportunities found on a local level could be targeted for intervention or service alterations, and a subsequent evaluation can determine if the changes were effective in reducing the missed opportunities.

Future work should explore the missed opportunities in different settings and why they happen. This requires comprehensive data collection that is representative of the population sampled. The data derived from such evaluations could help in understanding not only which missed opportunities have occurred but why they happen. This is important as data will be collected and reviewed to understand how care could be improved. By implementing such evaluations, areas of improvement could be identified and interventions and alterations to healthcare services can take place to improve patient care. There are, nevertheless, potential barriers, as some clinicians may feel that the care they deliver is of high quality and would be against objective scrutiny about the exact performance and evaluation of areas that need improvement. In addition, as some healthcare services are already limited in terms of resources in their ability to deliver care, identifying further areas that may require resources may be undesirable. However, it could be equally argued that improvements may be made in patient care to mitigate unnecessary costs but delivering improved quality of care.

## 5. Conclusions

In conclusion, in this manuscript, we explore the novel concept of missed opportunities related to the treatment of patients with HF within a structured framework of patient pathways. As patients with HF may be managed by a variety of professionals, some of whom may not be experts on HF guidelines and care, we show that there may be different missed opportunities along the patient journey or pathway of care. Future real-world evaluations are needed to determine, in the population of interest, that there are indeed significant missed opportunities in the treatment of patients with HF and what these missed opportunities may be, which could include those that are related to basic HF medications, specialist HF medications, etiology-specific therapy, device therapy, cardiac transplant, palliative care, and general opportunities, such as fluid status optimization, comorbidity optimization, education and self-management, mobility and lifestyle factors, social issues, and cardiac rehabilitation.

## Data Availability

Not applicable.
